# A Rare Case of Metastasis to the Thyroid Gland from Renal Clear Cell Carcinoma 11 Years after Nephrectomy and Concurrent Primary Esophageal Carcinoma

**DOI:** 10.1155/2018/3790106

**Published:** 2018-04-02

**Authors:** Mohammad Saud Khan, Veena Balakrishnan Iyer, Neha Varshney

**Affiliations:** ^1^Department of Internal Medicine, University of Toledo Medical Center, Toledo, OH 43614, USA; ^2^Department of Hematology and Oncology, Rhode Island Hospital, Providence, RI, USA; ^3^Department of Oncology, University of Toledo Medical Center, Toledo, OH 43614, USA; ^4^Department of Pathology, University of Toledo Medical Center, Toledo, OH 43614, USA

## Abstract

Renal cell carcinoma is known to cause metastasis to unusual sites, which can be both synchronous or metachronous. Thyroid gland is a rare site for metastasis, but when it occurs, renal cell carcinoma is the most common primary neoplasm. We report the case of a 81-year-old female patient who had a significant medical history of right clear cell renal carcinoma with adrenal metastasis. She underwent right radical nephrectomy and adrenalectomy followed by radiofrequency ablation of left adrenal metastasis and systemic chemotherapy with sunitinib. Eleven years later, she presented with dysphagia and was found to have distal esophageal adenocarcinoma. On imaging, there was incidental detection of a left renal mass lesion and a right thyroid nodule, which on histopathology and immunohistochemistry were confirmed to be clear cell carcinoma of renal origin.

## 1. Introduction

Thyroid gland is a rare site for clinically detectable metastasis despite having rich blood supply [1]. Renal cell carcinoma (RCC) is the most common primary neoplasm that metastasizes to thyroid gland and accounts for more than 50% of clinically recognized cases [2]. Other neoplasm frequently identified with metastasis to thyroid gland includes breast, lung, skin, and colon cancers. Metastasis accounts for 2-3% of all thyroid malignancies detected clinically [3, 4]. However, the incidence of thyroid metastasis has been reported to be higher (ranging from 1.9 to 22.4%) on autopsy studies [5]. We report the case of a 81-year-old female patient who developed recurrence of RCC with thyroid metastasis after 11 years following nephrectomy of initial tumor.

## 2. Case Report

**Figure 1:**
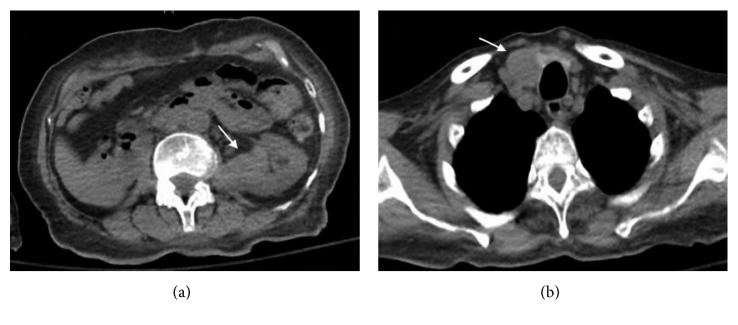
(a) Axial CT scan of the abdomen showing a mass lesion involving the inferior pole of the left kidney (arrow), breaching the renal capsule and infiltrating into adjacent retroperitoneal space. (b) Axial CT scan of the chest at the level of thyroid showing a well-defined hypodense nodule in the right lobe of the thyroid gland (arrow).

**Figure 2:**
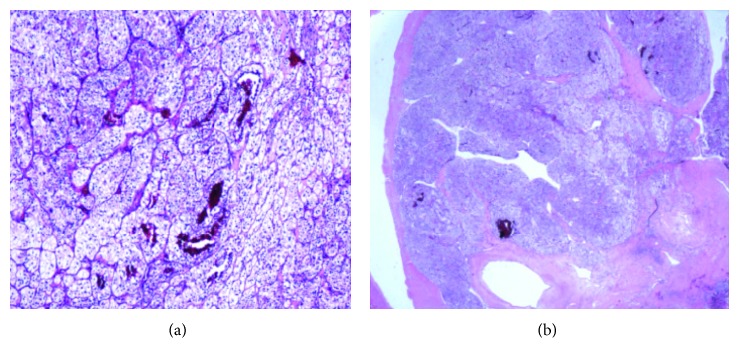
(a) Clear cells with increased vasculature consistent clear cell carcinoma of the kidney (40x, H&E). (b) Clear cell carcinoma invading renal vein (10x, H&E).

**Figure 3:**
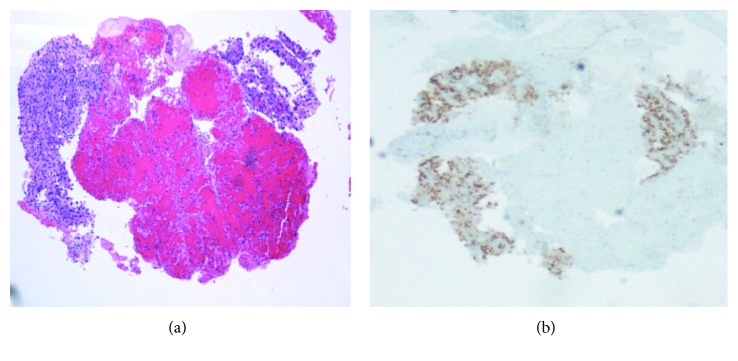
(a) Cell block (4x, H&E) of the thyroid showing atypical cells, which are PAX-8 positive (b) consistent with metastasis of renal origin.

A eighty-one-year-old African American female patient presented to the hospital with the complaints of dysphagia and loss of appetite for past few weeks. She had a past medical history of right RCC, clear cell carcinoma subtype with bilateral adrenal metastasis (T3b, N0, and M1) diagnosed in May 2006. At that time, she was treated with right radical nephrectomy and right adrenalectomy followed by radiofrequency ablation of left adrenal metastasis and systemic chemotherapy with sunitinib. She tolerated the treatment well with adequate control of her malignant disease for 11 years. Recently, in June 2017, she presented with the complaints of dysphagia predominantly for solid food, loss of appetite, and generalized fatigability. Physical examination was unremarkable. Esophagogastroduodenoscopy with endoscopic ultrasound showed a distal esophagus mass causing high-grade stricture extending up to the adventitia with no mediastinal lymphadenopathy. Metallic esophageal stent was placed in, and biopsies were obtained which showed adenocarcinoma of esophagus (T3N0M0). Computed tomography (CT) of the chest and abdomen showed distal esophageal thickening along with incidental findings of solid mass lesion involving the inferior pole of the left kidney measuring 3.4 × 2.6 cm in the largest dimension (Figure 1) and a well-defined hypodense nodule in the right lobe of the thyroid gland measuring 2.1 × 2.8 cm in the largest dimension (Figure 1). Biopsy of renal mass was done which showed neoplastic cells with clear cytoplasm arranged in nests and mitotic figures suggesting clear cell carcinoma (Figure 2). Fine-needle aspiration (FNA) from the thyroid nodule also identified neoplastic clear cells on cytology raising possibility of metastasis from RCC (Figure 3). This was confirmed with immunohistochemical stain, which showed atypical cells to be positive for PAX8 (Figure 3) and CAIX while negative for TTF-1. Positron emission tomography (PET) scans showed increased uptake in the distal esophagus, left renal mass, and right thyroid nodule. A diagnosis of concurrent distal esophageal adenocarcinoma along with left renal RCC recurrence and thyroid metastasis was made. The patient was planned for left nephrectomy and thyroidectomy. However, the patient wished to opt out for any surgical intervention or aggressive medical therapy and preferred to be treated with comfort care measures. The patient was treated with palliative intention and died 2 months later.

## 3. Discussion

RCC accounts for approximately 3-4% of all adult malignancies [6]. It is the most common renal malignancy and the second most common malignancy of urological tract [6, 7]. RCC is more common in males compared to females (ratio of 2 : 1) and occurs predominantly in the 6th to 8th decade of life with a median age of 64 years [6]. Major histopathological subtypes include clear cell carcinoma, papillary carcinoma, chromophobe carcinoma, collecting duct carcinoma, medullary carcinoma, and unclassified categories [8]. Clear cell carcinoma is the most common subtype making up to 75% of cases of RCC [9]. RCC is known to metastasize in unpredictable manner. The metastasis may be detected at the time of diagnosis (synchronous) or may be found years after the diagnosis and treatment (metachronous) [7]. The most common route of metastasis is hematogenous and likely involves lung, liver, bone, lymph nodes, adrenal gland, and brain. Head and neck metastasis are less frequent and of which thyroid is the most commonly involved site. Late recurrences and distant metastasis ranging from few months to several years after initial diagnosis are a notable feature of RCC. It has been estimated that 20–30% of patients including those who have undergone nephrectomy with curative intent will develop recurrence and out of these 50% will relapse distantly [7, 10]. Most of the recurrences are within 3 years of surgery, but delayed recurrences even after decades have been reported [11]. The longer the recurrence-free time from surgery, the more likelihood is of a true cure.

In majority of cases, thyroid metastasis is metachronous with average time of development being 9.4 years following resection of primary RCC [12]. But cases of RCC recurring with thyroid metastasis have been reported as late as 26 years [1]. These metastatic thyroid lesions may pose diagnostic challenge since they often occur years after treatment of primary lesion. RCC metastasis to thyroid can be asymptomatic and may be detected incidentally or may present with symptoms of palpable neck swelling, thyroid enlargement, dysphagia, dysphonia, or dyspnea [3]. Although metastasis to the thyroid gland may be suspected in patients with history of RCC, it is difficult to make a definitive preoperative diagnosis. Metastatic thyroid lesions usually appear as solid, hypoechoic, well-demarcated nodules with irregular border and increased vascularity on ultrasound imaging [13] and cold nodules on radioisotope uptake studies. These radiological features are nonspecific, and it is not possible to distinguish between primary and secondary thyroid neoplasms on imaging. FNA cytology serves as a reliable tool for establishing preoperative diagnosis. However, sometimes it is difficult to distinguish metastasis from tumors of thyroid, which can have clear cell component on FNA cytology alone. In these cases, immunohistochemistry is helpful and aids in differential diagnosis. Some of the traditional immunohistochemical markers for renal cell carcinoma are cytokeratin, vimentin, and CD10 [14]. However, recently novel markers for RCC have been identified which have increased sensitivity and specificity for identifying RCC. These include anti-carbonic anhydrase IX (CAIX), anti-human kidney injury molecule-1 (hKIM-1), and PAX8 [15]. The immunohistochemical markers used for identifying primary thyroid malignancies are thyroglobulin, thyroid transcription factor-1 (TTF-1), and calcitonin. In our case, immunohistochemical stains were positive for CAIX and PAX-8 and negative for TTF-1.

Definitive diagnosis of metastatic RCC is usually made by histopathological examination after thyroidectomy. Surgical resection with either partial or total thyroidectomy should be performed if thyroid gland is the only site for metastasis. Prognosis is good in this group [3, 16]. Patients with disseminated disease have poor prognosis and should undergo thyroidectomy only for palliation for compressive symptoms [16].

## 4. Conclusion

A thyroid nodule in a patient with a history of renal malignancy should be considered as potentially metastatic. Clinical manifestation and radiographic findings are nonspecific and are unable to distinguish between primary and secondary thyroid neoplasms. FNA cytology and immunohistochemistry are helpful in establishing diagnosis and should be obtained in suspected cases.
